# Camel tick species distribution in Saudi Arabia and United Arab Emirates using MaxEnt modelling

**DOI:** 10.1017/S0031182024001161

**Published:** 2024-08

**Authors:** Nighat Perveen, Sabir B. Muzaffar, Areej Jaradat, Olivier A. Sparagano, Arve L. Willingham

**Affiliations:** 1 Department of Biology, College of Science, United Arab Emirates University, Al-Ain, UAE; 2 Department of Veterinary Medicine, College of Agriculture and Veterinary Medicine, United Arab Emirates University, Al-Ain, UAE; 3 Department of Science, The Natural History Museum, London, UK; 4 Agricultural Sciences and Practice, Royal Agricultural University, Cirencester, UK; 5 Department of Infectious Diseases and Public Health, Jockey Club College of Veterinary Medicine and Life Sciences, City University of Hong Kong, Kowloon, Hong Kong SAR, China

**Keywords:** camel tick, *Hyalomma dromedarii*, MaxEnt, modelling, Saudi Arabia, species distribution, UAE

## Abstract

Ticks are important vectors and reservoirs of pathogens causing zoonotic diseases in camels and other livestock, rodents and other small mammals, birds and humans. *Hyalomma dromedarii* is the most abundant tick species in Saudi Arabia and United Arab Emirates (UAE) affecting primarily camels, and to a lesser extent, other livestock. Species presence data, land use/landcover, elevation, slope and 19 bioclimatic variables were used to model current and future distribution of *H. dromedarii* ticks using maximum entropy species distribution modelling (MaxEnt.). The model highlighted areas in the northern, eastern and southwestern parts of the study area as highly suitable for ticks. Several variables including land use/land cover (LULC) (53.1%), precipitation of coldest quarter (Bio19) (21.8%), elevation (20.6%), isothermality (Bio3) (1.9%), mean diurnal range [mean of monthly (max temp – min temp)] (Bio2) (1.8%), slope (0.5%), precipitation, seasonality (Bio15) (0.2%) influenced habitat suitability of ticks, predicting high tick density or abundance. Middle of the road scenario (ssp2-4.5) where CO_2_ levels remain similar to current levels, did not indicate a major change in the tick distributions. This tick distribution model could be used for targeting surveillance efforts and increasing the efficiency and accuracy of public health investigations and vector control strategies.

## Graphical abstract

**Figure:**
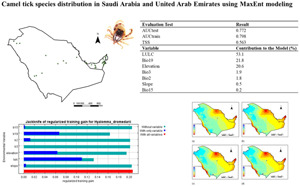

## Introduction

Ticks are haematophagous parasites that have great economic and ecological significance due to their capacity to transmit a variety of pathogens including viruses, bacteria and parasites to animals and humans. Expansion of the range of ticks due to rapid climate change carries profound threats for public health and society (Illoldi-Rangel *et al*., 2012; Rochlin and Toledo, 2020; Nuttall, 2022). Some of the most common tick-borne infections in the Middle East and North Africa (MENA) include Crimean–Congo haemorrhagic fever, anaplasmosis, theileriosis and babesiosis (Perveen *et al*., 2021*c*).

The 1-humped camel (*Camelus dromedarius*) is a highly valued species of livestock in Saudi Arabia and UAE (Gharbi *et al*., 2013). The current population of camels in UAE and Saudi Arabia is approximately 1 million (https://worldpopulationreview.com/country-rankings/camel-population-by-country). *Hyalomma dromedarii* ticks feed on the blood of camels and has been reported with high prevalence in the UAE (Perveen *et al*., 2020, 2021*b*; Perveen, 2021) and Saudi Arabia (Alanazi *et al*., 2018, 2020; Zakham *et al*., 2021). Crimean-Congo haemorrhagic fever (CCHF) is a deadly viral disease and virus transmitted by *Hyalomma* ticks (Perveen and Khan, 2022). In a systematic review of Crimean–Congo haemorrhagic fever in the Arab world (1978–2021), a total of 65 confirmed human cases have been reported from the 2 countries. Lately, *H. dromedarii* ticks were found positive for CCHF virus (CCHFV) in both UAE (Camp *et al*., 2020) and Saudi Arabia (Mohamed *et al*., 2017). In the MENA Region, *H. dromedarii* appears to be the vector of *Theileria annulata* where camels are raised together with cattle (Jacquiet *et al*., 1994), therefore, raising camels in mixed patterns could cause cross-infection in livestock (Tomassone *et al*., 2012). Furthermore, *H. dromedarii* can transmit various disease-causing pathogens for example, Dhori virus (Hoogstraal *et al*., 1981; Champour *et al*., 2016), the tropical theileriosis, *T. annulata* and *T. camelensis* (Hoogstraal *et al*., 1981; Hamed *et al*., 2011) Sindbis virus, Chick Ross and Kadam viruses (Al-Khalifa *et al*., 2007), *Coxiella burnetii* (Abdullah *et al*., 2018) and spotted fever rickettsia (Hoogstraal *et al*., 1981; Abdel-Shafy *et al*., 2012; Demoncheaux *et al*., 2012; Kernif *et al*., 2012; Kleinerman *et al*., 2013; Elzein *et al*., 2020).

Due to anthropogenic factors and climate change, tick-borne infectious diseases are increasingly becoming a significant public health threat (Gray *et al*., 2009). Ticks have unique physiological habits and spend their life cycle feeding on the host and in the habitat of the host. A range of environmental factors, such as substrate type, relative humidity and vegetation associated with the host habitat can affect tick abundance and distribution patterns (Ma *et al*., 2023). *Hyalomma dromedarii* may act as a 3-, 2- or 1-host species (Hoogstraal, 1956; Walker *et al*., 2003) and engorged female burrows a few centimetres under the ground to lay eggs in suitable microhabitats to avoid desiccation of eggs and new larvae (Alahmed and Kheir, 2003). Environmental factors and host range may help in the assessment of risk factors determining the distribution of tick-borne pathogens. Increased temperatures may positively affect the survival and reproduction of ticks (Ma *et al*., 2023). For example, rising temperatures in temperate and cold environments contributes to faster nymph maturation and shorter life cycles that increase tick abundance and also extend the period of ticks' host-seeking activity (Gray *et al*., 2009), thus encourage range expansion through establishment in new geographical ranges. Furthermore, global climate change not only influences tick distribution and abundance, but also affects tick-borne pathogen transmission by impacting land use, vegetation cover and distribution, and the abundance of reservoir hosts (Gray *et al*., 2009). Consequently, it is crucial to assess the current and future distribution of ticks for better management of tick-borne pathogens.

MaxEnt is a widely used technique in species distribution modelling because of its compatibility with presence-only (PO) data (Merow *et al*., 2013; Bradie and Leung, 2017; Phillips *et al*., 2022). Its algorithm is known for its robustness (Phillips et al., 2006) and outperforms many other PO modelling methods (Phillips *et al*., 2008; Wisz *et al*., 2008; Elith *et al*., 2011; Merow *et al*., 2013). MaxEnt was developed specifically for low sample-size data (PO locations) for multiple species (Phillips *et al*., 2006). This model used extensively to determine the distribution of numerous arthropods such as hard ticks and soft ticks that transmit important pathogens (Table 1).

**Table 1.** MaxEnt model used to predict the distribution of some hard and soft tick species

Tick species	References
*Hyalomma asiaticum, Dermacentor nuttalli*, *Ixodes persulcatus*, *Dermacentor silvarum*	Hu *et al*. (2022); Ma *et al*. (2023)
*Ixodes scapularis*	Johnson *et al*. (2016); Burrows *et al*. (2022); Zhang *et al*. (2022)
*Hyalomma marginatum*	Celina *et al*. (2023); Hekimoglu *et al*. (2023)
*Amblyomma testudinarium, Haemaphysalis flava Neumann, Haemaphysalis kitaokai Hoogstraal, Haemaphysalis longicornis Neumann, Haemaphysalis megaspinosa Saito, Ixodes ovatus Neumann*	Doi *et al*. (2021)
*Argas persicus, Dermacentor marginatus, Haemaphysalis concinna, Haemaphysalis longicornis, Ixodes granulatus, Rhipicephalus microplus, Rhipicephalus sanguineus sensu, Rhipicephalus turanicus*	Yang *et al*. (2020)
*Ornithodoros hermsi*	Sage *et al*. (2017)
*Amblyomma americanum*	Raghavan *et al*. (2016)
*Ixodes ricinus, Rhipicephalus annulatus, Dermacentor marginatus, Haemaphysalis punctata*	Williams *et al*. (2015)
*Otobius megnini*	Estrada-Peña *et al*. (2010)

In the MENA region, camel husbandry has increased in recent years with rapid economic development (Abahussain *et al*., 2002). In addition, climatic conditions of Saudi Arabia and UAE provide favourable conditions for tick species that are adapted to dry environments. Human activities and international trade increase the risk of tick expansion into new geographic zones. The present study was conducted to use MaxEnt modelling and the ArcGIS spatial technology platform to describe the current and predicted future distribution of the camel tick, *H. dromedarii* using occurrences of ticks in Saudi Arabia and UAE for the monitoring and surveillance of tick-borne pathogens associated with this species in the region.

## Materials and methods

### Collection and preparation of *H. dromedarii* occurrence data

Geo-referenced location points on ticks from Saudi Arabia and UAE from various resources including field collections and information from prior publications (Supplementary Table S1) were compiled. For Saudi Arabia, a literature review was conducted by search engines, Google Scholar, PubMed and Web of Science databases using the keywords ‘Saudi Arabia’, ‘tick’, ‘ticks’ and ‘tick-borne pathogens’ for articles published in the last 10 years. Only full-length research articles were used in this study. Review articles, letters to editors, short reports/communications, abstracts and conference proceedings were excluded. Literature containing geographical distribution information of camel tick species was filtered and extracted for their geographical location coordinates, Al-Khurma, Al-Kharj, Al-Hasa, Al-Qassim, Riyadh, Hail area, Amman Road, Madinah Road, Duba Road, Industrial area and Taif (Alanazi *et al*., 2018, 2020, 2021; Alreshidi *et al*., 2020; Zakham *et al*., 2021; Al Thabiani *et al*., 2022). After removing duplicates, only research articles were selected that provided coordinates for tick locations. Locations were chosen on the basis of presence of both hosts and tick vectors in the area. Occurrence data was rarefied using the spatially rarefy occurrence data tool in SDM toolbox in ArcGIS ver. 10.8.1 at a resolution of 15 km to avoid model over fitting and bias. It resulted in 32 occurrences which were later used in the spatial modelling.

### Collection and preparation of key variables influencing tick distribution

For the current model, land use/land cover (LULC), elevation, slope and 19 bioclimatic variables (Table 2) were selected initially. The bioclimatic variables and the DEM were obtained from WorldClim database (version 2.1) at ~1 km^2^ resolution (Fick and Hijmans, 2017) covering the period 1970–2000. Slope was calculated from elevation using the slope tool in ArcGIS. LULC was obtained from the European Space Agency climate change initiative for the year 2020 at 300 m resolution (Defourny *et al*., 2023). The extent and resolution of all selected variables were harmonized to the same study area size and resolution of ~1 km^2^ matching the bioclimatic variables using spatial analyst toolbox in ArcGIS.

**Table 2.** Environmental layers for species distribution models

Code	Bioclimatic variables
Bio1	Annual mean temperature
Bio2	Mean diurnal range [mean of monthly (max temp – min temp)]
Bio3	Isothermality (difference in day-to-night temperature oscillations in relation to annual temperature oscillations).
Bio4	Temperature seasonality [standard deviation × 100]
Bio5	Max temperature of warmest month
Bio6	Min temperature of coldest month
Bio7	Temperature annual range
Bio8	Mean temperature of wettest quarter
Bio9	Mean temperature of driest quarter
Bio10	Mean temperature of warmest quarter
Bio11	Mean temperature of coldest quarter
Bio12	Annual precipitation
Bio13	Precipitation of wettest month
Bio14	Precipitation of driest month
Bio15	Precipitation seasonality [coefficient of variation]
Bio16	Precipitation of wettest quarter
Bio17	Precipitation of driest quarter
Bio18	Precipitation of warmest quarter
Bio19	Precipitation of coldest quarter

Multicollinearity was assessed between the environmental variables using variance inflation factor analyses (VIF) in R (version 4.3.0). Highly correlated variables were eliminated considering a VIF <5 as a critical threshold (Akinwande *et al*., 2015). Variables that did not demonstrate any significant contribution to the model were subsequently removed (Redon and Luque, 2010) and 7 variables were finally selected for modelling: LULC, elevation, slope, mean diurnal range (Bio2), isothermality (Bio3), precipitation seasonality (Bio15) and precipitation of coldest quarter (Bio19). The contribution of each variable is assessed by utilizing jackknife tests to visualize variable significance and calculating the percentage contributions of the variables.

To assess the impact of different climate change scenarios on the spatial distribution of *H. dromedarii*, we excluded LULC. The future scenario used was ssp2-4.5, model CanESM5 which is among the most sensitive models in climate equilibrium (Swart *et al*., 2019) and covered the periods 2021–2040 and 2041–2060. The ssp2-4.5 Scenario is considered to be ‘middle of the road’ in which CO_2_ emissions remain approximately close to current levels before diminishing by the 2050s without achieving net-zero level emissions, and mean temperatures increase to 2.7°C by the end of the century. Socioeconomic factors continue to remain similar to historic trends and progress towards sustainability is slow, with development and income growing unevenly.

Bioclimatic variables were obtained from WorldClim database (version 2.1) at 30-arc-second (~1 km) resolution (Fick and Hijmans, 2017) except elevation and slope as it remained unchanged throughout the duration of the study. The extent and resolution of all selected variables were harmonized to the same study area size and resolution of ~1 km^2^ matching the bioclimatic variables using spatial analyst toolbox in ArcGIS. All variables were processed to have the same spatial extent and resolution of ~1 km^2^ using spatial analyst toolbox in ArcGIS.

### Maxent modelling procedures and calibration

For the modelling analyses, MaxEnt 3.4.3 (Phillips *et al*., 2022) was utilized. To address species-specific conditions and research objectives and to avoid relying solely on MaxEnt as a ‘blackbox’ tool (Hernandez *et al*., 2006; Phillips *et al*., 2008; Merow *et al*., 2013), we used the spatial jackknifing tool within the SDM toolbox in ArcGIS (Brown *et al*., 2017). This approach allowed for consideration of biological factors and provided a more comprehensive analysis. The tool tests the model using varying parameters and independently evaluates feature class parameters and the regularization multiplier (RM) to produce a model with the best performance. The RM enhances the model's predictive accuracy and achieve maximum entropy or a more uniform distribution which reduces model overfitting (Hernandez *et al*., 2006; Phillips *et al*., 2008). In addition, a bias file was generated using a gaussian kernel density approach for the sampling localities within the SDM toolbox. This bias file considers any sampling bias by providing MaxEnt with a background file that exhibits a similar level of bias observed in the presence localities. The bias file also enables the model to regulate the density and locations of background points, thereby avoiding the inclusion of less informative points that fall outside the known species range (Brown *et al*., 2017).

The final 7 environmental variables along with 32 presence points were used to run 10 replicates by the cross-validation method. An RM of 5 with linear features and cross-validation method for all analyses. Iterations were raised to 5000 to prevent under- or overprediction of spatial relationships, considering the recommended convergence threshold of 10^−5^. The bioclimatic model was then projected onto 2040 and 2060 years under ssp2-4.5 scenario with no extrapolation. Recognizing the limitation in the predictive capabilities of the modelling algorithm during projection (Merow *et al*., 2013), MaxEnt was prevented from extrapolating. In other words, MaxEnt did not make predictions beyond its training data during projecting. In addition, the provided bias file makes MaxEnt avoid sampling habitat outside the species' known occurrence (Brown *et al*., 2017).

### Model evaluation/performance assessment

To assess the model's performance, the receiver operating characteristic (ROC) curve was utilized, and the area under the curve (AUC) was calculated as a threshold-independent measure. AUC values range from 0 to 1, with higher values indicating better model performance (Merckx *et al*., 2011). For a threshold-dependent measure, the true skill statistics (TSS) method was employed, using the threshold of maximum training sensitivity and specificity (West *et al*., 2016). TSS accounts for both omission and commission errors and is less influenced by prevalence (Allouche *et al*., 2006). TSS was interpreted based on ranges: <0.4 poor, 0.4–0.8 useful and >0.8 good-to-excellent performance (Zhang *et al*., 2015). TSS calculations were conducted using Microsoft Excel.

To determine the relative importance of each variable in the model, contribution percentage and jackknife analysis were conducted using MaxEnt. Response curves were also measured for each predictor variable to illustrate the changes in habitat suitability corresponding to varying levels of the environmental variables.

### Model exploration between current and future variables

To examine the differences between current and future variables, multivariate environmental similarity surfaces (MESS) and the most dissimilar variable (MoD) of the MESS map were computed using MaxEnt (Elith *et al*., 2010). MESS shows similarity of a given point in the future to its reference current set of environmental layers. MoD shows the variable with the smallest similarity at each point (Elith *et al*., 2010). Limiting factor analyses (LF) were also conducted in MaxEnt to examine the most influencing variable on model prediction at each point for current and future predictions (Elith *et al*., 2010). All maps were processed and visualized in ArcGIS.

## Results

### Model evaluation and sensitivity analysis

**Figure 1.**
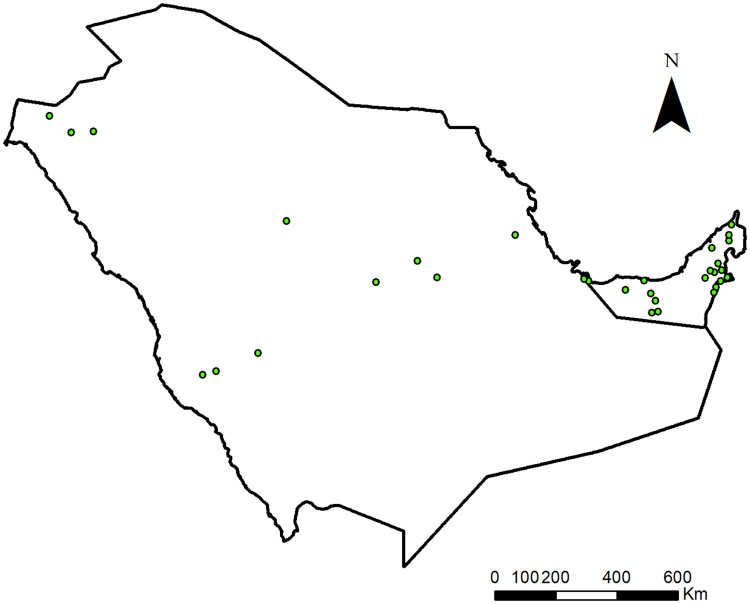
*Hyalomma dromedarii* occurrence points in Saudi Arabia and UAE used for the distribution modelling.

**Figure 2.**
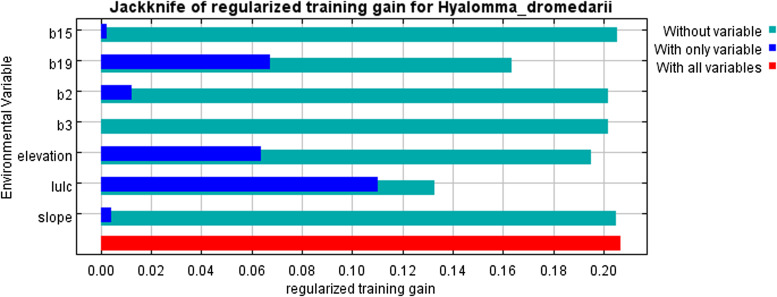
Jackknife test (evaluation of each variable significance). LULC (land use/land cover), Bio2 (mean diurnal range), Bio3 (isothermality), Bio15 (precipitation seasonality), Bio19 (precipitation of coldest quarter).

The current distribution map of *H. dromedarii* is given in Fig. 1 (Supplementary Table S1). The distribution model showed consistent spatial distribution, with AUC-test at 0.772 and AUC-train at 0.798, meaning the model had 77.2% performance (Table 1). The TSS result also indicated that the model is useful as the averaged value was TSS = 0.563 (Table 3) (Zhang *et al*., 2015). LULC, Bio19 and elevation were the top contributors to the model, with 53.1, 21.8 and 20.6% (Table 3), respectively. The jackknife test revealed that when LULC was used in isolation, the environmental variable showed the highest gain (Fig. 2) and this variable when omitted decreased the gain. The second most significant variable was the Bio19 when not used in the model, dropped the average gain followed closely by elevation (Fig. 2).

**Table 3.** Evaluation test, sensitivity test and each variable contribution percentage in the model of *Hyalomma dromedarii*

Evaluation test	Result
AUCtest	0.772
AUCtrain	0.798
TSS	0.563
Variable	Contribution to the model (%)
LULC	53.1
Bio19	21.8
Elevation	20.6
Bio3	1.9
Bio2	1.8
Slope	0.5
Bio15	0.2

### Predicted areas of *Hyalomma dromedarii* with potential suitability

**Figure 3.**
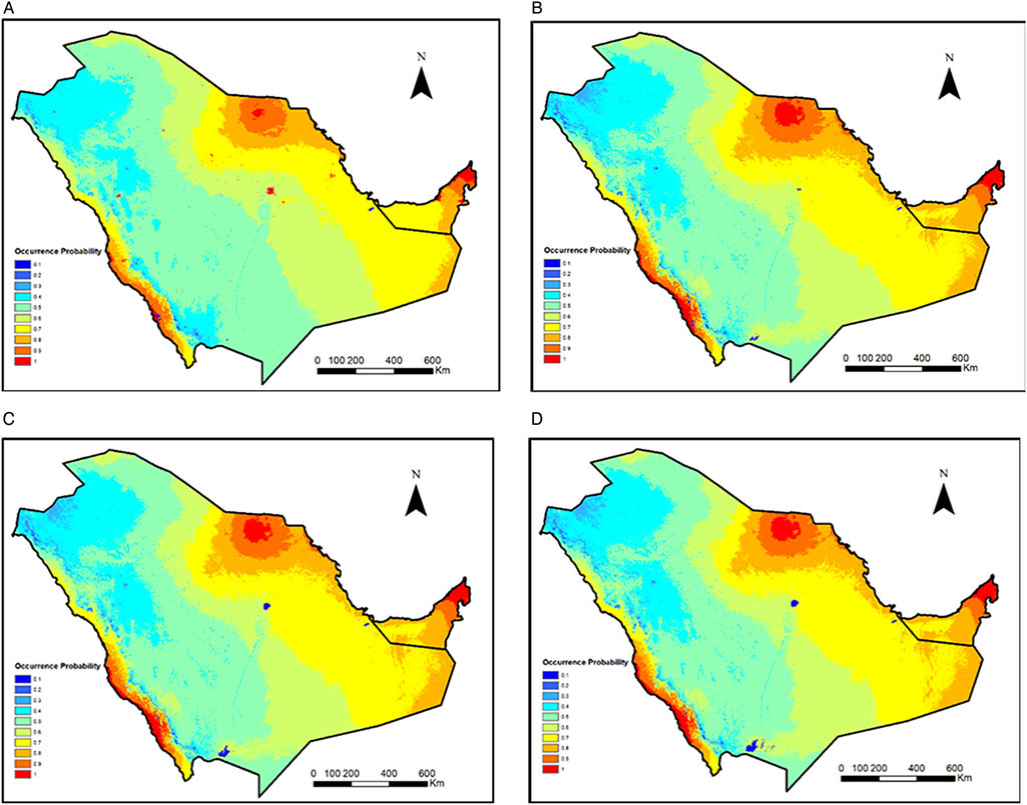
Geographic distribution of *Hyalomma dromedarii* for (a) current, (b) current based on bioclimatic variables, (c) year 2040 under ssp2-4.5 scenario and (d) year 2060 under ssp2-4.5 scenario both based on bioclimatic variables.

Highly suitable areas (>0.6) existed mostly in the northern and eastern parts of the study area, with a considerable area near the Red Sea coast in the south (Fig. 3a, b). Very highly suitable areas (>0.8) corresponded mostly with urban cities across the study area (Fig. 3a, b). The UAE ranged from high (western region) to very high (mid- and northern region) in suitability. Highly suitable areas covered a good portion of Saudi Arabia and extended from north to east. Moderately suitable areas were mainly centred in the western and mid-regions of Saudi Arabia.

The potential future distribution is not predicted to change dramatically under the ssp2-4.5 scenario for the years 2040 and 2060 (Fig. 3c, d). A noticeable change is seen in Riyadh and Najran cities where suitability decreased sharply.

### Model exploration

**Figure 4.**
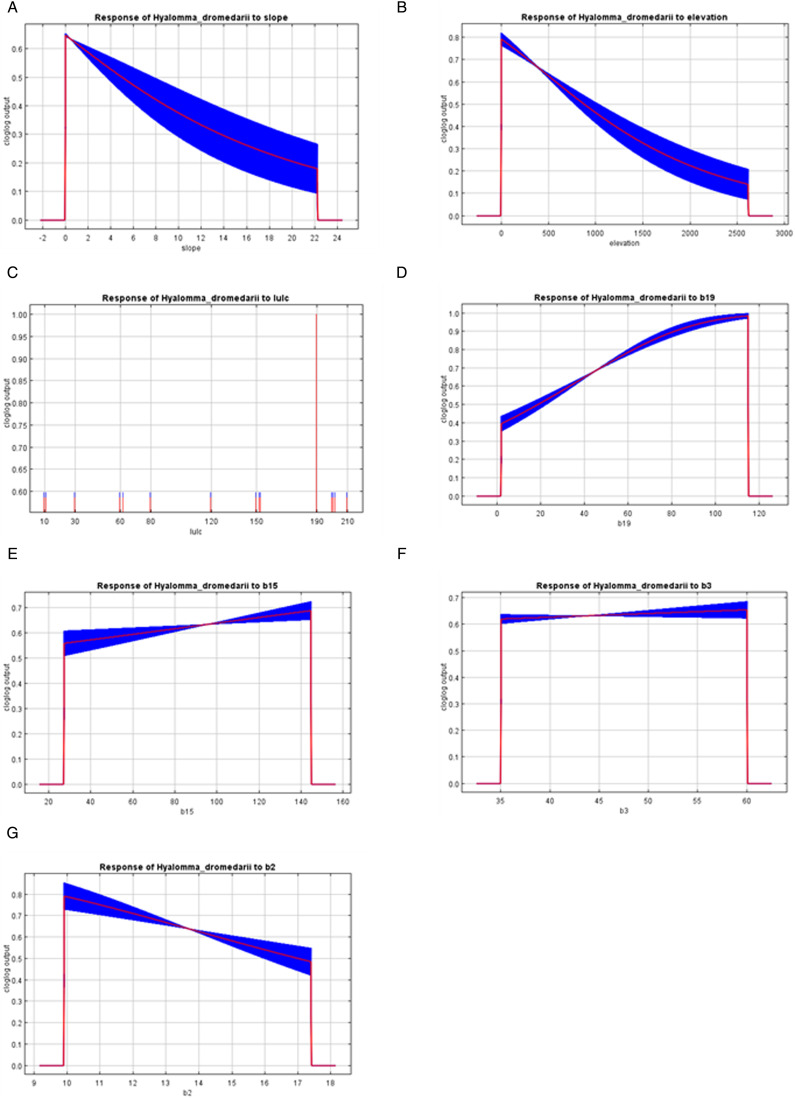
Environmental variables response curves display probable occurrence of *Hyalomma dromedarii*. (a) Slope (m), (b) elevation (m), (c) LULC (land use/land cover), (d) Bio19 (precipitation of coldest quarter, mm), (e) Bio15 (precipitation seasonality, per cent), (f) Bio3 (isothermality, percent), (g) Bio2 (mean diurnal range, °C).

With the increasing value of slope, elevation and Bio2 (mean diurnal range) variables, the probability of occurrence declined sharply (Fig. 4a, b, g). High suitability (>0.6) occurred in areas where elevation ⩽500 m and b2 ⩽14.5°C. In contrast, potential suitability increased with increasing Bio19 (precipitation of coldest quarter) with highly suitable areas existing in areas receiving ⩾38 mm (Fig. 4d). Suitability also slightly increased with increasing Bio15 (precipitation seasonality) and Bio3 (isothermality) (Fig. 4e, f). For LULC (land use/land cover) variable, suitability was moderate for croplands (10, 30), tree cover (60, 80), shrubland (120), sparse vegetation (150) and high for urban areas (190) (Fig. 4c).

**Figure 5.**
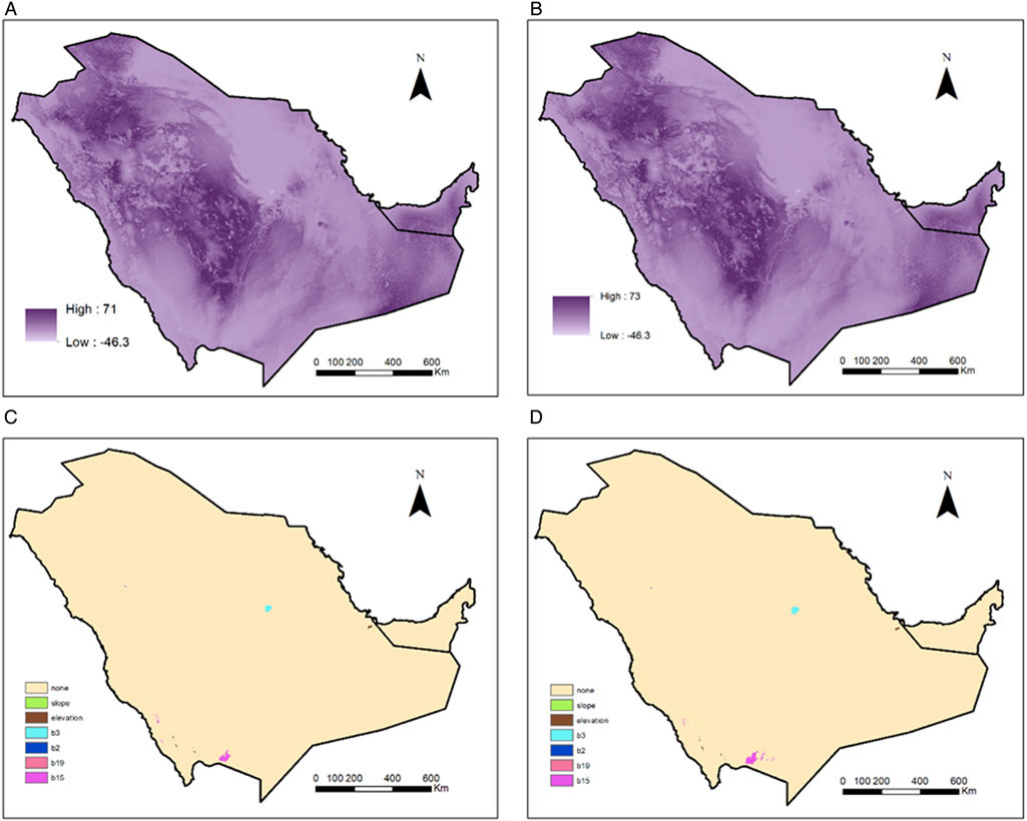
Model maps: (a, b) MESS (multivariate environmental similarity surfaces) analysis presenting the degree of resemblance between future and current set of environmental layers; (c, d) most dissimilar variable (MoD) analysis. (a, c) year 2040 and (b, d) year 2060, all under the bioclimatic distribution model.

The MESS analysis values ranged from −46 to 71 and from −46 to 73 for the years 2040 and 2060, respectively (Fig. 5a, b). Most areas were similar with varying degrees to current environmental conditions. Some areas had negative values demonstrating a degree of newness in environmental space. The highest novelty was in Riyadh city followed by Najran city where the novelty was mostly driven by Bio3 and Bio15 in both years, respectively (Fig. 5c, d).

**Figure 6.**
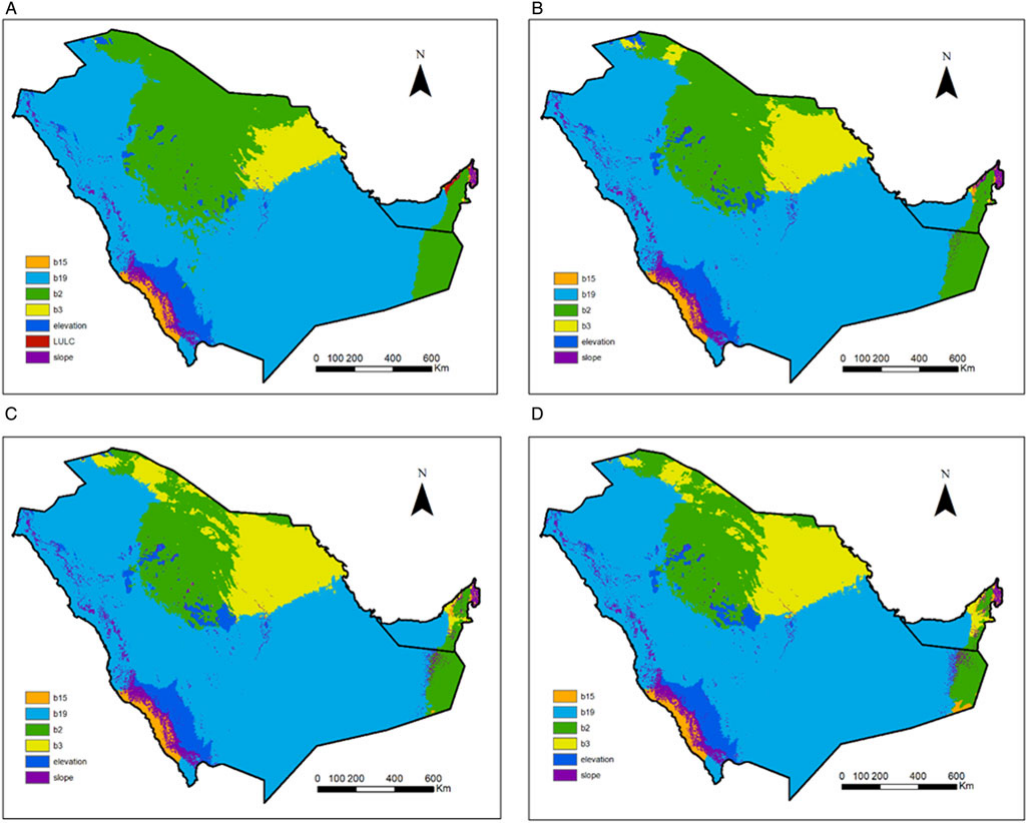
Limiting factor (LF) analyses highlighting the most influencing variable on model prediction for (a) current, (b) current, based on bioclimatic variables, (c) year 2040 under ssp2-4.5 scenario and (d) year 2060 under ssp2-4.5 scenario both based on bioclimatic variables.

Bio19 is the main limiting factor over the predicted existing range, followed by Bio2 in the limiting factor analyses (Fig. 6a, b). Southern region in Saudi Arabia had elevation, slope and Bio15 slope as the limiting factors. Similarly, northern areas in the UAE had slope and Bio15 as the limiting factors. LULC was also limiting the distribution on the northern west coast of the UAE (Fig. 6a). For the potential future distribution (Fig. 6c, d), Bio3 limiting effect increased over Bio2 in northern Saudi areas as well in eastern UAE areas. Elevation limiting effect also increased in middle Saudi areas.

## Discussion

The MaxEnt model has been widely used for predicting distributions of hundreds of animal species (Elith *et al*., 2006). Our model helped us to better understand the environmental niche of *H. dromedarii* tick species in Saudi Arabia and UAE. The predicted maps developed from this model on current occurrences of *H. dromedarii* with a high probability based on suitable environmental conditions. The modelled distribution of *H. dromedarii* indicated that highly suitable areas existed mostly in the northern, eastern and southern portions of the study range. In the UAE, the eastern to northern regions were classified as highly suitable for ticks. These regions include portions of eastern Abu Dhabi emirate, including Al Ain, bordering Oman, various cities within north-eastern portions of Abu Dhabi, such as Mafrak, Dubai and the northern Emirates, where the coastal zones influence moisture and temperature profiles. This increases the suitability of ticks in these regions. In addition, farms abound with high density of camels and other livestock that help to sustain tick populations (Perveen *et al*., 2021*c*). In Saudi Arabia, areas that were highly suitable were within the north-central region, the eastern edge south of the UAE and the southwestern shoreline bordering the Red Sea, whereas western and mid-regions were of moderate suitability for ticks. The north central regions with high suitability were east of the Al Nafud desert region. This area is adjacent to areas that are around 500 m in elevation, and receives more precipitation, making them suitable for ticks. Similarly, the southwestern regions are immediately adjacent to the Asir Mountain range that has elevations of over 2000 m, with significant moisture draining out of them into the Red Sea, increasing moisture content and influencing temperature along the southwestern coastline. Saudi Arabia and UAE camel and livestock farming represent an essential habitat for *H. dromedarii* and other ticks, which are likely enhanced in these regions due to better environmental conditions compared to the remaining parts of the study area.

Bioclimatic factors combined with LULC have a cumulative influence on determining the suitability of tick habitats. The survival of ticks during its off-host phase is heavily reliant on variables like temperature and humidity (Apanaskevich *et al*., 2013; Pascoe *et al*., 2019). The elevation of an area affects its microclimate, the presence of hosts and the vegetation. Additionally, slope serves as an indicator of subsurface water flow velocity, runoff rate and soil moisture content (Pascoe *et al*., 2019). The presence and characteristics of vegetation also impact the suitability of tick habitats. Vegetation plays a crucial role in the life cycle of tick hosts, such as camels, where the availability of cropland, herbaceous cover and water are decisive factors for host presence in a given area (Apanaskevich *et al*., 2013; Pascoe *et al*., 2019). Based on the current climate, several variables influenced the *H. dromedarii* tick distribution including land use/land cover LULC (53.1%), Bio19 (21.8%), elevation (20.6%), Bio3 (1.9%), Bio2 (1.8%), slope (0.5%) and Bio15 (0.2%). The precipitation of coldest quarter is significant to the tick survival in the winter season. For land use/land cover variable, suitability was modest for sparse vegetation (150) and high for urban areas (190), maybe due to anthropogenic factors such as land-use change, agriculture practices, forest fragmentation or urbanization. The jackknife results showed that the LULC when used in isolation was with the highest gain. The high suitability/high tick density sites are the areas categorized by a high proportion of land cover (Burrows *et al*., 2022). Moreover, the land-use and land cover patterns may provide microclimatic conditions through vegetation covers that serve as ticks habitats (Doi *et al*., 2021; Khwarahm, 2023). Our findings are almost similar to the one conducted in Iraq on distribution of the *Hyalomma* spp. where distribution influenced by LULC (50.8%), followed by elevation (30.4%) (Khwarahm, 2023). A similar study was conducted on distribution of *Ornithodoros hermsi* where annual temperature range (Bio7) contribution was highest (18.9%) in the model, followed by elevation (18.1%), and precipitation of the warmest quarter (Bio18) in Sage *et al*. (2017). However, in another study minimum temperature of coldest month (Bio6) and precipitation of driest quarter (Bio17) strongly influenced the model (Porretta *et al*., 2013). In Mongolia, annual precipitation (Bio12) and elevation influenced the *Hyalomma asiaticum* distribution (Ma *et al*., 2023). The differences in the impact of environmental variables in other studies are most likely due to species-specific niche requirements, an area that requires extensive study in the Middle East region.

Response curves of the environmental variables showed that *H. dromedarii* potential suitability increased with increasing precipitation of coldest quarter (Bio19), in areas receiving ⩾38 mm while declining sharply with the increasing value of slope and elevation. The potential future distribution is not predicted to change dramatically under the ssp2-4.5 scenario for 2040 and 2060 years; however, a visible change has been seen in Riyadh and Najran cities where suitability decreased sharply mostly driven by isothermality and precipitation seasonality in both years, respectively. Therefore, climate change can impact the tick distribution in forthcoming years. This species disperses naturally with the help of infested animals, but it can also inhabit new ranges through travel and transportation of animals across the borders. Due to its high prevalence on camels in both countries (Alanazi *et al*., 2020; Perveen *et al*., 2020; Perveen, 2021), its distribution is a continued threat in the region. In addition, it serves as a reservoir of many tick-borne pathogens. Therefore, the current study will assist researchers and health care managers to devise the strategies to limit the distribution of ticks to better avoid tick-borne zoonotic diseases in the future. Various zoonotic pathogens have been reported previously in the region including MERS-CoV (Zaki, 2012; de Groot *et al*., 2013; Perveen *et al*., 2021*a*) and CCHFV (Camp *et al*., 2020; Perveen and Khan, 2022) posing a serious threat to camel farming and human and animal health. *Hyalomma* spp. are primary vectors of CCHFV (Perveen and Khan, 2022). Recently, *H. dromedarii* tested positive for CCHFV in both countries (Mohamed *et al*., 2017; Camp *et al*., 2020). Previously, a virus related to the tick-borne encephalitis complex has also been detected in Saudi Arabia (Zaki, 1997).

The true distribution of ticks and other invertebrates are difficult to determine as widespread sampling of ticks, hosts and their environments are not available in the MENA region (Perveen *et al*., 2021*c*). In the absence of true distribution, model validation provides the best way of assessing if the predicted distribution falls within reasonable bounds of statistical uncertainty (Convertino *et al*., 2014; Liu *et al*., 2016; Chen *et al*., 2019). We used AUC and TSS values that indicated that the model was able to predict the distribution based on the quality of the data available. The Kappa statistic is typically used for validating the accuracy of the distribution models, including MaxEnt models (Allouche *et al*., 2006). However, the Kappa statistic has a linear relationship with prevalence (the proportion of sites in which the species is present), making it a potential statistical artefact (Allouche *et al*., 2006; Liu *et al*., 2016). In comparison, the TSS is independent of prevalence making it better in accurately predicting distribution (Allouche *et al*., 2006; Liu *et al*., 2016). Values of TSS that we obtained were in the useful category. This may suggest that further data from more sampling sites could improve the model.

In summary, the model showed that LULC, precipitation of coldest quarter, and elevation were most influential for predicting the areas as highly suitable for ticks. Other environmental variables which contributed in the model were isothermality, mean diurnal range [mean of monthly (max temp – min temp)], slope, precipitation seasonality. The model presented here provides valuable information on camel tick species distribution in Saudi Arabia and UAE. The predicted distribution of *H. dromedarii* may allow researchers and health officials to conduct risk assessments targeting specific pathogens and potentially reduce the chance of outbreaks through surveillance and mitigation efforts.

## Supporting information

Perveen et al. supplementary material Perveen et al. supplementary material

## Data Availability

Locations used in this study are available in supplementary material.

## Supplementary material

The supplementary material for this article can be found at https://doi.org/10.1017/S0031182024001161.
