# Supplementary material for: Camel tick species distribution in Saudi Arabia and United Arab Emirates using MaxEnt modelling
Source: Parasitology. 2024 Dec 19;151(9):1024–34. doi: 10.1017/S0031182024001161 (PMC11772089; doi:10.1017/S0031182024001161)
Supplement: Perveen et al. supplementary material [file S0031182024001161sup001.docx]

Table S1: The distribution points of *Hyalomma dromedarii* in Saudi Arabia (KSA) and UAE for model operation.

| Species | Longitude | Latitude | Region |
| --- | --- | --- | --- |
| *Hyalomma_dromedarii* | 55.67471 | 24.345929 | UAE |
| *Hyalomma_dromedarii* | 55.5081 | 23.84347 | UAE |
| *Hyalomma_dromedarii* | 55.47016 | 24.28937 | UAE |
| *Hyalomma_dromedarii* | 55.454876 | 23.698655 | UAE |
| *Hyalomma_dromedarii* | 55.191872 | 24.119656 | UAE |
| *Hyalomma_dromedarii* | 55.649861 | 24.027389 | UAE |
| *Hyalomma_dromedarii* | 55.853694 | 24.137333 | UAE |
| *Hyalomma_dromedarii* | 55.34334 | 24.33066 | UAE |
| *Hyalomma_dromedarii* | 55.576157 | 24.552465 | UAE |
| *Hyalomma_dromedarii* | 53.3903615 | 24.0520875 | UAE |
| *Hyalomma_dromedarii* | 53.6004325 | 23.666667 | UAE |
| *Hyalomma_dromedarii* | 53.7305984 | 23.4532655 | UAE |
| *Hyalomma_dromedarii* | 53.8099693 | 23.1322992 | UAE |
| *Hyalomma_dromedarii* | 53.6247635 | 23.1078102 | UAE |
| *Hyalomma_dromedarii* | 52.851032 | 23.771787 | UAE |
| *Hyalomma_dromedarii* | 51.7712051 | 24.0294038 | UAE |
| *Hyalomma_dromedarii* | 51.6303332 | 24.0962654 | UAE |
| *Hyalomma_dromedarii* | 55.3879229 | 25.0046087 | UAE |
| *Hyalomma_dromedarii* | 55.8947183 | 25.2204731 | UAE |
| *Hyalomma_dromedarii* | 55.9686383 | 25.6859011 | UAE |
| *Hyalomma_dromedarii* | 55.8881901 | 25.382115 | UAE |
| *Hyalomma_dromedarii* | 40.406156 | 21.275094 | KSA |
| *Hyalomma_dromedarii* | 42.033333 | 21.916667 | KSA |
| *Hyalomma_dromedarii* | 47.3 | 24.133333 | KSA |
| *Hyalomma_dromedarii* | 49.6 | 25.383333 | KSA |
| *Hyalomma_dromedarii* | 42.866667 | 25.8 | KSA |
| *Hyalomma_dromedarii* | 45.5 | 24 | KSA |
| *Hyalomma_dromedarii* | 46.716667 | 24.633333 | KSA |
| *Hyalomma_dromedarii* | 35.9 | 28.883333 | KSA |
| *Hyalomma_dromedarii* | 37.183333 | 28.433333 | KSA |
| *Hyalomma_dromedarii* | 36.533333 | 28.4 | KSA |
| *Hyalomma_dromedarii* | 40.8 | 21.383333 | KSA |
